# Worldwide measurements of bioturbation intensity, ventilation rate, and the mixing depth of marine sediments

**DOI:** 10.1038/s41597-019-0069-7

**Published:** 2019-05-13

**Authors:** Martin Solan, Ellie R. Ward, Ellen L. White, Elizabeth E. Hibberd, Camilla Cassidy, Jasmin M. Schuster, Rachel Hale, Jasmin A. Godbold

**Affiliations:** 1 Ocean and Earth Science, National Oceanography Centre Southampton, University of Southampton, Waterfront Campus, European Way, Southampton, SO14 3ZH UK; 2 Department of Ocean Sciences, Memorial University of Newfoundland, St. John’s, Newfoundland, NL Canada

**Keywords:** Element cycles, Ecological modelling, Ecosystem ecology, Behavioural ecology

## Abstract

The activities of a diverse array of sediment-dwelling fauna are known to mediate carbon remineralisation, biogeochemical cycling and other important properties of marine ecosystems, but the contributions that different seabed communities make to the global inventory have not been established. Here we provide a comprehensive georeferenced database of measured values of bioturbation intensity (Db, n = 1281), burrow ventilation rate (q, n = 765, 47 species) and the mixing depth (L, n = 1780) of marine soft sediments compiled from the scientific literature (1864–2018). These data provide reference information that can be used to inform and parameterise global, habitat specific and/or species level biogeochemical models that will be of value within the fields of geochemistry, ecology, climate, and palaeobiology. We include metadata relating to the source, timing and location of each study, the methodology used, and environmental and experimental information. The dataset presents opportunity to interrogate current ecological theory, refine functional typologies, quantify uncertainty and/or test the relevance and robustness of models used to project ecosystem responses to change.

## Abstract (summary)

**Table:** 

Design Type(s)	data integration objective • species comparison design
Measurement Type(s)	Publication
Technology Type(s)	digital curation
Factor Type(s)	temporal_instant • geographic location • depth • Species • season
Sample Characteristic(s)	Polychaeta • Earth (Planet) • marine benthic biome • Malacostraca • Bivalvia • Echinoidea • Ophiuroidea

Machine-accessible metadata file describing the reported data (ISA-Tab format)

## Background & Summary

Marine sediments are known to harbour significant levels of biodiversity that play a key role in biogeochemical cycling, carbon storage and the regulation of climate active gases^1–3^, but the geographic contribution of extant benthic communities is not well constrained at large scales^4^. Quantifying the extent, timing and way in which organisms transport particles and pore water fluids^5^ has received a considerable amount of attention^6^, yet few attempts to seek universalities, generalities, and particularities have taken place that can inform the architecture of global biogeochemical models^7–10^. Variations in the intensity of faunal mediation in relation to changing conditions that alter species interactions^11^, community structure^12^ and environmental setting^13–15^ are well-known and mean that the contributions of individual species and/or definable communities cannot be applied universally^16^, yet these sources of variation are not generally incorporated into modelling frameworks^17^. Indeed, most models are parameterised with broad functional descriptors or selected values of bioturbation that oversimplify or misrepresent temporal and spatial variation in the mediating role of biota^18–20^, largely because comprehensive compilations of such biological information are not readily available^21^. For these reasons, the treatment of key processes can differ greatly between models such that simulated ecosystem outcomes commonly misalign with ecosystem properties measured at local to regional scales^17^, frustrating efforts to accurately project the effects and consequences of environmental change^22^.

Descriptions of how infaunal invertebrates mediate ecosystem properties are common in the literature and have largely become synonymous with particle displacement and burrow ventilation^23^, although alternative descriptors have been considered and emphasised^24^. As sediment particle reworking often consists of a series of small particle displacement events, standard practice has been to treat the resulting vertical profile of mixing in an analogous way to that of diffusive heat transport, calculating a biodiffusion coefficient (Db, cm^2^ year^−1^) that describes the rate at which the variance of the location of a particle tracer changes over time within the sediment profile^25^. Similarly, as the active transfer of fluid by infaunal organisms may be orders of magnitude greater (volumetrically) than particle reworking^26^, the non-diffusive exchange of pore-water solutes with over-lying water is routinely examined^27^, but these data have not previously been collated in an accessible archive. The combined effect of particulate and fluid transport on sediment biogeochemical processes is reflected in the vertical colour transition (from brown to olive green/black) of the sediment profile^28^, dictated by the transition from iron (oxyhydr)oxides at the surface to black sulphidic phases at depth^29^ that correlate with a variety of environmental drivers^30^. Hence, regions of high reflectance (brown) in an image represent a well-mixed region of sediment and provide a reasonable approximation of the mixing depth^31^.

Here, motivated by the need to relate changes in ecosystem properties to local heterogeneity rather than global mean conditions^17,32^, we have collated the extensive repository of information that exists in the primary scientific literature concerning how faunal communities redistribute sediment particles, ventilate their burrows and effect the depth to which mixing typically occurs in relation to their physical location. Our hope is that the inherent spatial and temporal heterogeneity shown within these data will be embraced by modellers, statisticians and ecologists and contribute to the development of next generation biogeochemical models that can better inform conservation and management strategies.

## Methods

We searched the Thomson Reuters Web of Science collection (http://www.webofknowledge.com, accessed 07/03/2019) using a ‘General Search’ across all databases with the search term (i) *bioturbation*, (ii) sediment profile imag*, and (iii) *bioirrigation OR burrow ventilation* in the titles and key words of all document types, in all languages, for the publication years 1864 to 2018. Citation returns were manually searched for reported values of the sediment mixing depth (L_,_ cm)^30,31^, the biodiffusion coefficient (D_b_, cm^2^ year^−1^) estimated from models of sediment particle reworking^6,25^, and the rate of ventilation (q, ml h^−1^ ind.^−1^) for named macro-invertebrate species or mixed communities. These data for L and D_b_ supersede records collated elsewhere^7–10^ and include observations from the older literature (pre-1970) cited by the authors of the returns from our search.

For each unique record, we collated associated environmental metadata (latitude, longitude, water depth, sedimentation rate), information on the methodology used, and details about the timing (year, season, month) and ecoregion (following accepted biogeographical typologies)^33,34^ from the original publication, personal communication with the corresponding author and/or from third party sources of information. Where specific values were not presented in the original publication and had to be derived, values were extracted from graphical summaries using *Web Plot Digitiser* (https://automeris.io/WebPlotDigitizer/). When the location of a study was not provided, latitude and longitude coordinates and/or water depth were retrieved from Google Earth (http://earth.google.com/) and manually cross referenced with site descriptions within the source publication. Following standard practice^9^, the seasonal offset between Northern (NH) and Southern (SH) hemisphere was corrected by attributing a nominal season to each study: Spring, April-June in the NH or October–December in the SH; Summer, July–September in the NH or January–March in the SH; Autumn, October–December in the NH or April–June in the SH; or Winter, January–March in the NH or July–September in the SH. Due to variations in seasonal timing at any given latitude, the scheme is not necessarily representative of geographical clines in forcing. Data collected from multiple months or unspecified periods are also included. The methodology used to generate each record includes 21 techniques for L and D_b_ (reviewed in ref.^35^) and 18 techniques for q (reviewed in ref.^36^).

As species ventilation behaviour varies over time^37^, we distinguish ventilation measurements based solely on active bouts of ventilation (q1, an indication of peak activity) from those estimated over extended periods of time that span rest periods (q2, a more representative indication of species contribution). Similarly, in recognising that experimental configuration^24,38^ and the geometry of the sediment-water interface^39^ can influence species behaviour, our database includes information on aquaria dimensions. Given the time span of the studies under consideration, species nomenclature has been standardised in line with the *World Register of Marine Species*^40^.

## Data Records

**Fig. 1:**
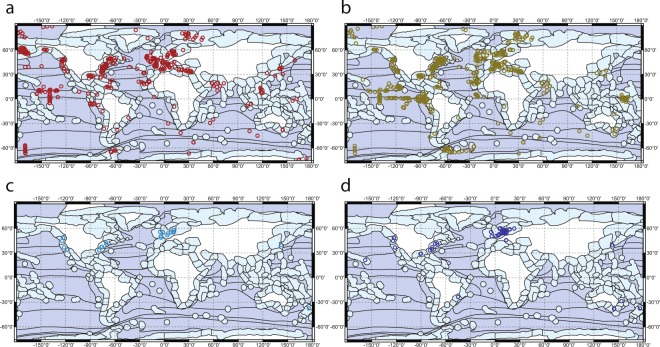
The geographical location of reported bioturbation parameter values. (**a**) D_b_, the biodiffusion coefficient, (**b**) L, the sediment mixing depth, (**c**) q1, the ventilation rate for named macro-invertebrate species or for mixed communities taken during active bouts of ventilation and (**d**) q2, the ventilation rate for named macro-invertebrate species or for mixed communities estimated over extended periods of time. Data points may represent multiple observations at that locality. The boundaries of ecoregion domains and divisions^33^ (dark blue shading) and provinces^34^ (light blue shading) are indicated.

Data records are available via an unrestricted repository hosted by Harvard Dataverse^41^. Data represent reported values for the biodiffusion coefficient (D_b_, cm^2^ year^−1^; Fig. 1a) and/or the sediment mixing depth (L, cm; Fig. 1b) for specific locations and can be found in solan_etal_DbL.csv. Separately, the data set also includes volumetric ventilation flow rates (q, ml h^−1^ ind.^−1^) for named macro-invertebrate species or mixed communities taken during active bouts of ventilation (q1, Fig. 1c) and/or estimated over extended periods of time (q2, Fig. 1d) for specific locations. These can be found in solan_etal_q.csv. The number of records within the dataset are listed to ecoregion (Table 1), method of quantification (Table 2) and by season and water depth (Table 3). Table 3 also includes the number of experimental observations of q1 and q2 listed against taxonomic class. A summary of the definitions for the descriptors (=column headings) used in the Db and L (Descriptor categories S1) and q (Descriptor categories S2) datasets are documented separately in solan_etal_suppl_info_v3.docx^41^.

**Table 1:** Number of observations for bioturbation intensity (Db), mixing depth (L) and ventilation rate (q1 and q2) listed by marine realm.

Realm	Db	L	q1	q2
Arctic	68	45	0	0
Central IndoPacific	17	48	0	0
Eastern IndoPacific	29	4	0	7
Not allocated (Polar)	4	4	0	0
Not allocated (Temperate)	12	17	0	0
Not allocated (Tropical)	36	21	0	0
Southern Ocean	58	17	0	0
Temperate Australasia	3	5	3	13
Temperate Northern Atlantic	661	1312	206	553
Temperate Northern Pacific	272	122	17	45
Temperate South America	32	25	0	6
Temperate Southern Africa	1	1	0	0
Tropical Atlantic	33	41	0	0
Tropical Eastern Pacific	36	88	0	0
Western IndoPacific	19	30	0	0
**Grand total**	**1281**	**1780**	**226**	**624**

**Table 2:** Number of observations for bioturbation intensity (Db), mixing depth (L) and ventilation rate (q1 and q2) categorised against method of quantification.

Method	Db	L	q1	q2
^137^Cs	43	10	0	0
^14^C	2	59	0	0
^210^Pb	551	429	0	0
^222^Ra	10	10	0	0
^228^Th	12	0	0	0
^234^Th	423	46	0	0
^235^Th	4	1	0	0
^239240^Pu	34	16	0	0
^32^Si	3	3	0	0
^7^Be	39	8	0	0
calculated	0	71	0	0
Chla	71	7	0	0
Bromide	0	0	10	155
Caesium	0	0	0	4
clearance	0	0	0	34
Doppler	0	0	14	23
dye	0	0	19	28
Eh	0	109	0	0
Em	0	0	95	46
estimate	0	0	1	0
glassbeads	19	0	0	0
hydraulic	0	0	6	6
luminescence	0	2	0	0
luminophores	57	21	0	0
model	0	0	0	6
OrgC	0	1	0	0
oxygen	0	0	6	6
permeability	0	0	1	0
pet	0	0	0	7
piv	0	0	7	2
pressure	0	0	48	265
radon	0	0	11	0
SPI	5	833	0	0
TCO_2_	0	0	0	3
tekbeads	8	8	0	0
thermistor	0	0	6	38
uranine	0	0	2	0
visual	0	104	0	0
xray	0	42	0	0
**Grand total**	**1281**	**1780**	**226**	**623**

Method definitions are listed as Descriptor categories S1 and S2 in in solan_etal_suppl_info_v3.docx^41^.

**Table 3:** Number of observations for bioturbation intensity (Db), mixing depth (L) and ventilation rate (q1 and q2) for season and depth category.

	Db	L	q1	q2
**Season**				
Spring	295	332	81	125
Summer	266	607	28	115
Autumn	245	233	3	9
Winter	70	31	41	70
Multiple	68	72	1	78
Total:	944	1275	154	397
**Depth category**				
0–50 m	277	956	225	599
50–200 m	281	276	0	16
200–1000 m	245	145	0	9
1000–4000 m	303	251	0	0
4000–6000 m	175	152	0	0
>6000 m	0	0	0	0
Total:	1281	1780	225	624
**Class**				
Bivalvia	—	—	9 [4]	22 [5]
Echinoidea	—	—	5 [2]	10 [1]
Ophiuroidea	—	—	0 [0]	9 [1]
Malacostraca	—	—	37 [9]	89 [11]
Polychaeta	—	—	157 [11]	427 [21]
Community	—	—	18 [n/a]	67 [n/a]
Total:			226	624

For q, the number of experimental observations are listed against taxonomic class. The number of species considered within each taxonomic class are indicated in square brackets.

## Technical Validation

The data has been collated from the peer-reviewed literature (Data Source S1 in solan_etal_suppl_info_v3.docx)^41^ and has undergone rigorous quality control prior to publication. Each individual record (unique identification number) in the dataset is traceable to the point of origin (data source identification number)^41^.

## Usage Notes

We have included all reported values from the literature without prejudice or downstream processing steps. Reporting errors and updates of the data will be periodically issued. Users should use the latest version of the data listed (under the ‘versions’ tab) at Harvard Dataverse^41^ and maintained at *Bioturbation Online* (http://bioturbation.online). This contribution is based on data release 3.0. There are no limitations on the use of these data.

## ISA-Tab metadata file

Download metadata file
